# Homologous recombination deficiency and genomic alterations in advanced prostate cancer: insights for precision therapy

**DOI:** 10.1093/oncolo/oyag100

**Published:** 2026-03-24

**Authors:** Chiara Mercinelli, Dean Pavlick, Neeraj Agarwal, Philippe E Spiess, Roger Li, Ashish M Kamat, Petros Grivas, Shilpa Gupta, Brigida Anna Maiorano, Valentina Tateo, Antonio Cigliola, Michela Piacentini, Joseph M Jacob, Gennady Bratslavsky, Alina Basnet, Jeffrey S Ross, Andrea Necchi

**Affiliations:** Vita-Salute San Raffaele University, Via Olgettina 60, Milan, 20132, Italy; Department of Medical Oncology, IRCCS San Raffaele Hospital, Comprehensive Cancer Center, Via Olgettina 60, 20132, Milan, Italy; Foundation Medicine Inc, Cambridge, MA, 02141-2115, United States; Department of Medical Oncology, Huntsman Cancer Institute, University of Utah, Salt Lake City, UT, 84112, United States; Department of Genito-Urinary Oncology, H. Lee Moffitt Cancer Center and Research Institute, Tampa, FL, 33612, United States; Department of Genito-Urinary Oncology, H. Lee Moffitt Cancer Center and Research Institute, Tampa, FL, 33612, United States; Department of Urology, MD Anderson Cancer Center, Houston, TX, 77030, United States; Fred Hutchinson Cancer Center, University of Washington, Seattle, WA, 98109, United States; Cleveland Clinic Taussig Cancer Institute, Cleveland, OH, 44195, United States; Department of Medical Oncology, IRCCS San Raffaele Hospital, Comprehensive Cancer Center, Via Olgettina 60, 20132, Milan, Italy; Department of Medical Oncology, IRCCS San Raffaele Hospital, Comprehensive Cancer Center, Via Olgettina 60, 20132, Milan, Italy; Vita-Salute San Raffaele University, Via Olgettina 60, Milan, 20132, Italy; Department of Medical Oncology, IRCCS San Raffaele Hospital, Comprehensive Cancer Center, Via Olgettina 60, 20132, Milan, Italy; Department of Medical Oncology, IRCCS San Raffaele Hospital, Comprehensive Cancer Center, Via Olgettina 60, 20132, Milan, Italy; Section of Innovation Biomedicine-Oncology Area, Department of Engineering for Innovation Medicine (DIMI),University of Verona and University Hospital Trust (AOUI), Verona, 37124, Italy; Department of Urology, Upstate Medical University, Syracuse, NY, 13210, United States; Department of Urology, Upstate Medical University, Syracuse, NY, 13210, United States; Division of Hematology-Medical Oncology, Upstate Cancer Center, Syracuse, NY, 13210, United States; Foundation Medicine Inc, Cambridge, MA, 02141-2115, United States; Department of Urology, Upstate Medical University, Syracuse, NY, 13210, United States; Vita-Salute San Raffaele University, Via Olgettina 60, Milan, 20132, Italy; Department of Medical Oncology, IRCCS San Raffaele Hospital, Comprehensive Cancer Center, Via Olgettina 60, 20132, Milan, Italy

**Keywords:** prostate cancer, precision medicine, homologous recombination, BRCA

## Abstract

**Background:**

the homologous recombination deficiency signature (HRDsig) is emerging as a novel potential predictor of PARP-inhibitor (PARPi) response. We compared genomic alterations (GA) across BRCA2-loss, BRCA2 short variant–mutated (svmut), and BRCA2–wild type (wt) clinically advanced prostate carcinoma (CAPC) samples, combined with an assessment of HRDsig status to gain a better understanding of these biomarkers.

**Methods:**

Comprehensive genomic profiling (CGP) was performed on 22 061 CAPC cases to evaluate all classes of GA. Microsatellite instability (MSI) status, tumor mutational burden (TMB), genomic ancestry, were derived from sequencing data. HRDsig status was calculated using genome-wide copy number features. PD-L1 expression was assessed by IHC. Comparisons were performed using Fisher’s exact test with Benjamini–Hochberg correction for false discovery.

**Results:**

Among 22 061 CAPC cases, 10.2% were HRDsig+. HRDsig+ and were enriched for *BRCA2, RB1, MYC, RAD21*, and *AR* alterations, while *SPOP*, MSI-high, high TMB, and MMR signatures were more frequent in HRDsig− cases. Across BRCA2-defined subgroups, 597 (2.7%) were BRCA2-loss, 1085 (4.9%) BRCA2-svmut, and 20 379 (92.4%) BRCA2-wt. Both BRCA2-loss and BRCA2-svmut were associated with higher GA burden and enrichment for *RB1* alterations. BRCA2-loss cases displayed lower TMB-high incidence, while BRCA2-svmut showed higher MSI-high and TMB-high incidence. Most *BRCA2* alterations were bi-allelic, with concurrent alterations in other HRR genes being rare.

**Conclusions:**

BRCA2-loss CAPC displays a distinct genomic landscape, marked by robust HRD features, suggesting the potential of higher sensitivity to PARPi. These findings highlight the relevance of HRDsig, and routinely use of CGP in refining patient selection for PARPi and guiding the design of future clinical trials.

Implications for Practice This study highlights the clinical relevance of HRDsignature (HRDsig) and *BRCA2*-loss as complementary biomarkers in clinically advanced prostate cancer (CAPC).

HRDsig identifies a distinct subgroup of tumors with robust homologous recombination deficiency, beyond *BRCA* status alone, and may improve selection of patients most likely to benefit from PARP inhibitors. Moreover, the genomic differences observed between *BRCA2*-loss and other types of *BRCA2* alterations underscore the biological heterogeneity within *BRCA2*-mutated tumors and reinforce the importance of comprehensive genomic profiling to refine treatment selection and guide future biomarker-driven clinical trials.

## 1. Introduction

Clinically advanced prostate carcinoma (CAPC) is a complex and heterogeneous malignancy, frequently associated with poor prognosis and limited therapeutic options, particularly in the castration-resistant stages.^1^^,^^2^ Efforts to improve outcomes recently have focused on identifying useful biomarkers to guide more precise and personalized approaches.

Homologous recombination deficiency (HRD) is an established biomarker in many tumor types including CAPC, resulting from the dysfunction of homologous recombination repair (HRR) system, a crucial pathway for double-strand DNA breaks (DSBs) repair. Alterations in this pathway have become particularly significant in several malignancies including CAPC, since that poly (ADP-ribose) polymerase inhibitors (PARPi) demonstrated their efficacy in patients with alterations in HRR-related genes.^3^ HRR impairment in prostate cancer can be associated with biallelic *BRCA2* loss, which has been linked to prolonged sensitivity to PARPi, as it reduces the likelihood of resistance through *BRCA2* reversion mutations.^4^ Notably, *BRCA* alterations have also been consistently associated with worse prognosis, including higher tumor aggressiveness, earlier progression, and shorter overall survival in patients with CAPC.^5^

However, focusing only on *BRCA1/2* mutations can be limiting, because patients with other or no known HRR gene alterations may also benefit from PARPi, and many CAPC tumors with detectable HRR gene mutations may still have functional HRR system, highlighting the need for a comprehensive pan-tumor biomarker that better captures HRD status.

HRD signature (HRDsig) is a promising genomic biomarker aiming to identify tumors with HRD.^6^ Unlike traditional approaches that focus on specific gene mutations, HRDsig is based on a machine learning algorithm that analyses genome-wide patterns of copy number variations, including loss of heterozygosity, large-scale transitions, and telomeric allelic imbalances. This approach can detect the inability of HRR system to properly repair DSBs, identifying specific “genomic scars” associated with HRD phenotype.

In the present study, we applied comprehensive genomic profiling (CGP) to over 22 000 CAPC tumor samples to analyze a wide range of molecular features, including, but not limited to, HRR genomic alterations, microsatellite instability (MSI), tumor mutation burden (TMB), genomic ancestry, and PD-L1 expression, and to evaluate their associations with HRDsig status.

Our aim was to provide greater insight into the role of HRDsig in CAPC and evaluate its potential as a valuable tool in personalized therapeutic decision making.

## 2. Methods

### 2.1 Comprehensive genomic profiling

Hybrid capture-based next-generation sequencing (NGS) assays were performed on ≥50 ng of DNA extracted from 40 µm of formalin-fixed, paraffin-embedded (FFPE) clinically stage IV prostate cancer tumor samples in a Clinical Laboratory Improvement Amendments (CLIA)-certified and College of American Pathologists (CAP)-accredited laboratory (FMI, Cambridge, MA).

The FoundationOne and FoundationOne CDx assays were utilized to conduct CGP, allowing for the detection of diverse classes of genomic alterations.^7^ These include single-nucleotide variants (SNVs), insertions/deletions, genomic rearrangements, copy number amplifications, and homozygous deletions. The assays analyze 324 cancer-related genes and the intronic regions of 28 genes frequently involved in genomic rearrangements in cancer. Tumor Mutational Burden (TMB) was calculated from 0.80 Mb of sequenced DNA as the number of non-driver somatic coding mutations per megabase (mut/Mb) of the genome and categorized as “very high” if ≥20 mut/Mb, “high” if between 10 and 19 mut/Mb, and “low” if <10 mut/Mb.^8^ MSI was determined on at least 1500 loci.^9^ Tumor cell PD-L1 expression was determined by immunohistochemistry (Dako 22C3) and defined as tumor proportion score (TPS) positive if ≥1% and highly positive if ≥50%.

Genomic ancestry for each patient sample was determined using a SNP-based classifier to assign ancestral population groups (African, Admixed American, East Asian, European, and South Asian), as previously described.^10^ Germline status was predicted through a validated somatic-germline computational approach (somatic-germline-zygosity [SGZ]), applied exclusively to substitution and indel variant types. This method predicted the somatic or germline origin of variants, as well as their homozygous, heterozygous, or sub clonal state, based on deep massively parallel sequencing of cancer specimens without matched normal tissues.^11^ Additionally, genomic signature assignment was performed using trinucleotide signatures from the Catalogue of Somatic Mutations in Cancer (COSMIC) and attributed according to established computational methodologies.^12^

HRDsig is a machine learning algorithm designed to predict genomic scarring indicative of HRD.^6^ It leverages a comprehensive set of copy number features, including absolute modeled copy number, segment size, oscillation patterns, and the number of breakpoints per chromosome arm. These features are analyzed across the genome, with specific attention to the telomeric and centromeric regions of chromosome arms. HRDsig is expressed as a continuous score ranging from 0 to 1, with a predefined threshold of 0.7 used to classify a sample as HRDsig positive (HRDsig+). This cutoff was established to achieve 90% sensitivity in detecting biallelic *BRCA1/2* alterations in cancers often driven by *BRCA* mutations, such as ovarian, prostate, pancreatic, and breast cancers.

### 2.2 Statistical analysis

Statistical analyses were conducted using R software version 4.2.2. The Fisher’s exact test was used to compare proportions of categorical variables, while differences in continuous variables were assessed using the Wilcoxon rank-sum test. All *P*-values were two-sided, and multiple hypothesis testing was adjusted using the Benjamini-Hochberg procedure to determine the false discovery rate.^13^

## 3. Results

### 3.1 HRDsig status and genomic alterations

DNA was extracted and analyzed from a total of 22 061 CAPC samples, comprising 19 817 (89.8%) HRDsig− and 2244 (10.2%) HRDsig+ cases.

HRDsig+ patients were only slightly older than HRDsig− (median age 70 vs. 68; *P* < .0001; Table 1). Similarly, the median number of genomic alterations (GA) per tumor was higher in HRDsig+ cases (4 vs. 3, *P* < .0001). Genomic ancestry distribution was similar for European ancestry (76.6% in HRDsig+ vs. 74.8% in HRDsig−), while African ancestry was more frequent in the HRDsig− group (15.4% vs. 13.1%; *P* = .01).

**Table 1.** Clinical and genomic characteristics of CAPC stratified by HRD signature status.

	CAPC HRDsig−	CAPC HRDsig+	***P*-Value** ^a^
**Number of cases**	19 817	2244	/
**Median age**	68	70	<.0001
**Median GA/tumor**	3	4	<.0001
**AFR ancestry**	15.4%	13.1%	.0110
**EAS ancestry**	1.6%	2.2%	.0605
**EUR ancestry**	74.8%	76.6%	NS
**Pathogenic genomic alterations** ^b^			
***APC***	9.3%	6.6%	<.0001
***AR***	10.8%	17.3%	<.0001
***ATM***	5.9%	4.9%	.0648
***BRCA2***	3.3%	52.3%	<.0001
***CDK12***	5.5%	1.2%	<.0001
***MYC***	9.1%	19.2%	<.0001
***PIK3CA***	6.6%	5.4%	.0473
***PTEN***	32.0%	29.9%	.0648
***RAD21***	7.2%	16.3%	<.0001
***RB1***	4.5%	9.1%	<.0001
***SPOP***	11.1%	5.9%	<.0001
***TMPRSS2***	31.9%	30.0%	.0848
**Homozygous copy number deletion** ^b^			
***BRCA2***	0.6%	21.4%	<.0001
***PTEN***	22.2%	19.2%	.0009
**IO drug biomarkers**			
**MSI-high**	3.3%	0.5%	<.0001
**TMB ≥ 20 mut/Mb**	3.1%	0.5%	<.0001
**PD-L1 1-49% TPS**	11.2%	9.7%	NS
**MMRsig**	4.0%	2.0%	<.0001

This table compares the clinical, genomic, and biomarker features of CAPC cases stratified by HRD signature (HRDsig).

Abbreviations: GA = genomic alterations; AFR = African; EAS = East Asian; EUR = European; IO = Immuno-Oncology; MSI = Micro-satellite instability; TMB = tumor mutational burden; MMR = mismatch repair.

a False discovery rate (FDR) corrected using Benjamini/Hochberg adjustment.

b Genes only included if seen at >5% in any population.

**Figure 1.**
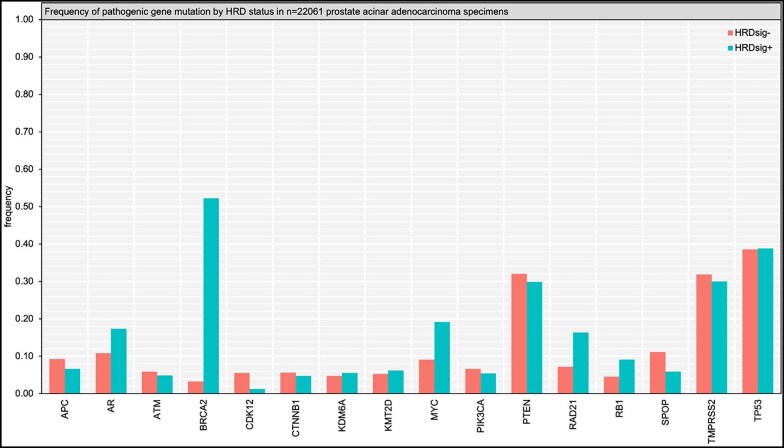
Bar chart showing the frequency of pathogenic gene mutation by HRD status in *n* = 220 061 prostate acinar adenocarcinoma specimens.

Pathogenic GA differed significantly between the two groups (Figure 1). While *BRCA1* GA were extremely rare in both groups, *BRCA2* alterations were substantially more frequent in HRDsig+ cases (52.3% vs. 3.3%, *P* < .0001). Higher rates of *MYC* (19.2% vs. 9.1%, *P* < .0001), *RAD21* (16.3% vs. 7.2%, *P* < .0001), *RB1* (9.1% vs. 4.5%, *P* < .0001), and *AR* (17.3% vs. 10.8%, *P* < .0001) were also observed in HRDsig+ group. Conversely, *APC* (9.3% vs. 6.6%, *P* < .0001) and *SPOP* (11.1% vs. 5.9%, *P* < .0001) alterations were more frequent in HRDsig− cases. No significant differences were observed for *ATM, PTEN*, or *TMPRSS2* alterations.

Bi-allelic homozygous copy number deletions were enriched in HRDsig+ cases for *BRCA2* (21.4% vs. 0.6%, *P* < .0001), while *PTEN* deletions were slightly more frequent in HRDsig− cases (22.2% vs. 19.2%, *P* = .0009). In the HRDsig− group, 89.9% of *BRCA1* and 48.9% of *BRCA2* mutated CAPC were mono-allelic.

In regards to immune-related biomarkers, MSI-high and TMB ≥ 20 mut/Mb were significantly more frequent in HRDsig− cases (3.3% vs. 0.5%, *P* < .0001, and 3.1% vs. 0.5%, *P* < .0001, respectively). PD-L1 expression (1%-49% TPS) showed no significant difference between groups (11.2% in HRDsig− vs. 9.7% in HRDsig+, NS). Finally, mismatch repair (MMR) signatures were slightly more prevalent in HRDsig− cases (4.0% vs. 2.0%, *P* < .0001).

### 3.2 Biomarker and genomic alteration (GA) findings in BRCA2-loss CAPC

A comparative analysis of clinical and genomic features was conducted across three groups within a cohort of 22 061 CAPC samples: (1) 597 patients (2.71%) with a homozygous deletion of *BRCA2* (hereafter referred to as *BRCA2*-loss), (2) 1085 (4.92%) with short variant sequence mutated (*BRCA2*-svmut), and (3) 20 379 (92.37%) with wild type *BRCA2* (*BRCA2*-wt).

Most *BRCA1/2* alterations identified were biallelic, primarily driven by high rates of homozygous deletions and biallelic *BRCA2* coding mutations. Bi-allelic inactivation was significantly more frequent in *BRCA2* compared to *BRCA1* (82% vs. 27%, *P* < .001). Additionally, co-occurring alterations in other HRR genes were rare in tumors with biallelic inactivation.

Median age was similar across the three groups (Table 2). The genomic ancestry distribution was comparable among the groups, with European ancestry consistently predominant (74%-76%). Both *BRCA2*-loss and *BRCA2*-svmut CAPC exhibited a higher frequency of GA per tumor compared to *BRCA2*-wt CAPC (*P* < .0001 for both comparisons).

**Table 2.** Clinical and genomic characteristics of CAPC by BRCA2 status.

	*BRCA2* loss	*BRCA2* SVmut	*BRCA2* wt	**BRCA2 loss vs. wt (*P*-Value** ^a^ **)**	**BRCA2 svmut vs. wt (*P*-Value** ^a^ **)**
**Number of cases**	569	1071	20181	/	/
**Median age**	68	68	68	1	.0682
**Median GA/tumor**	4	4	3	<.001	<.001
**Genomic ancestry**					
**AFR**	14.9%	14.4%	15.4%	1	.473
**AMR**	6.7%	6.6%	7.5%	.996	.441
**EAS**	2.8%	1.4%	1.6%	.109	.826
**EUR**	75.4%	76.4%	74.6%	1	1
**SAS**	0.2%	1.2%	0.9%	.206	.333
**Pathogenic genomic alterations**					
***AR***	9.7%	9.9%	11.7%	.201	.100
***ASXL1***	2.6%	7.6%	4.0%	.135	<.001
***ATM***	1.6%	4.3%	5.9%	<.001	.044
***BRAF***	1.6%	5.2%	4.1%	.003	.100
***BRCA2***	100.0%	100.0%	0.0%	0	0
***CDK12***	0.2%	2.4%	5.4%	<.001	<.001
***CTNNB1***	7.0%	6.1%	5.4%	.128	.349
***KMT2D***	5.6%	9.4%	5.1%	.589	<.001
***LYN***	3.5%	2.3%	5.%	.0482	<.001
***MSH2***	0.5%	5.4%	2.2%	.009	<.001
***MYC***	17.4%	15.6%	9.5%	<.001	<.001
***PIK3CA***	5.3%	8.3%	6.6%	.254	.050
***PTEN***	26.5%	22.1%	31.8%	.015	<.001
***RAD21***	26.0%	24.5%	12.3%	<.001	<.001
***RB1***	7.7%	10.0%	4.6%	.003	<.001
***SPEN***	1.1%	5.5%	1.9%	.237	<.001
***SPOP***	7.9%	6.8%	10.8%	.047	<.001
***TMPRSS2***	36.6%	22.9%	32.1%	.047	<.001
**TP53**	29.0%	28.7%	39.2%	<.001	<.001
**MSI-high**	0.2%	7.8%	2.8%	<.001	<.001
**Median TMB**	3.6	3.6	1.3	<.001	<.001
**TMB ≥ 10 mut/Mb**	2.1%	13.1%	3.9%	.079	<.001
**TMB ≥ 20 mut/Mb**	0.1%	8.0%	2.6%	<.001	<.001
**HRDsig positive**	87.4%	71.6%	8.2%	<.001	0
**COSMIC trinuclotide signature**					
**Alkylating**	0.0%	0.0%	0.0%	1	1
**APOBEC**	0.0%	0.1%	0.1%	1	1
**MMR**	1.9%	8.5%	3.5%	.109	<.001
**POLE**	0.0%	0.7%	0.0%	1	<.001
**Tobacco**	0.5%	0.4%	0.1%	.021	.013
**UV**	0.0%	0.2%	0.0%	1	.048
**PD-L1 DAKO 22C3 IHC**					
**PD-L1 low +**	8.1%	13.3%	11.1%	.51	.328
**PD-L1 high +**	0.6%	2.0%	0.8%	1	.067

This table provides a comprehensive comparison of clinical, genomic, and pathological features across three prostate cancer groups: BRCA2-loss (*n* = 569), BRCA2-svmut (*n* = 1071), and BRCA2 wt (*n* = 20 181).

a False discovery rate (FDR) corrected using Benjamini/Hochberg adjustment.

*BRCA2*-loss tumors were enriched for *TMPRSS2–ERG* fusions compared with *BRCA2*-wt (36.6% vs. 32.1%, *P* = .047), whereas SPOP mutations were less frequent (7.9% vs. 10.8%, *P* = .047). In addition, alterations in key DNA repair and tumor suppressor genes, such as *CDK12* (0.2% vs. 5.4%), *ATM* (1.6% vs. 5.9%), and *PTEN* (26.5% vs. 31.8%) were significantly less frequent in BRCA2-loss compared with *BRCA2*-wt (*P* < .0001, *P* < .001, *P =* .015, respectively). By contrast, *RB1* alterations were more common (7.7% vs. 4.6%, *P* < .001), potentially reflecting enrichment for neuroendocrine features in this subgroup.

In the *BRCA2*-svmut group, *TMPRSS2–ERG* fusions were less frequent compared with *BRCA2*-wt (22.9% vs. 32.1%, *P* < .0001). Similarly, *SPOP* mutations (6.8% vs. 10.8%, *P* < .0001), *CDK12* (2.4% vs. 5.4%, *P* < .001), and *ATM* (4.3% vs. 5.9%, *P* = .011) were less common compared to *BRCA2-*wt, while *RB1* mutations were enriched (10.0% vs. 4.6%, *P* < .001).

Although MSI-high status was rare across all groups, it was more frequent in *BRCA2*-svmut cases (7.8%) compared to *BRCA2*-loss (0.2%) and *BRCA2*-wt (2.8%). Similarly, tumors with TMB high were significantly more frequent in *BRCA2*-svmut cases (13.1% with ≥10 mut/Mb; 8.0% with ≥20 mut/Mb), whereas *BRCA2*-loss samples demonstrated usually low TMB (2.1% ≥10 mut/Mb; 0.1% ≥20 mut/Mb). PD-L1 expression showed no major differences across groups. Low-PD-L1 expression (TPS 1%-49%) ranged between 8.1% and 13.3%, while high PD-L1 expression (TPS ≥50%) was rare, observed in 0.6%-2.0% of cases.

The frequency of MMR-associated genomic signatures was significantly higher in both *BRCA2*-loss and *BRCA2*-svmut cases compared to *BRCA2*-wt. APOBEC- and POLE-associated signatures were extremely rare in all groups.

**Figure 2.**
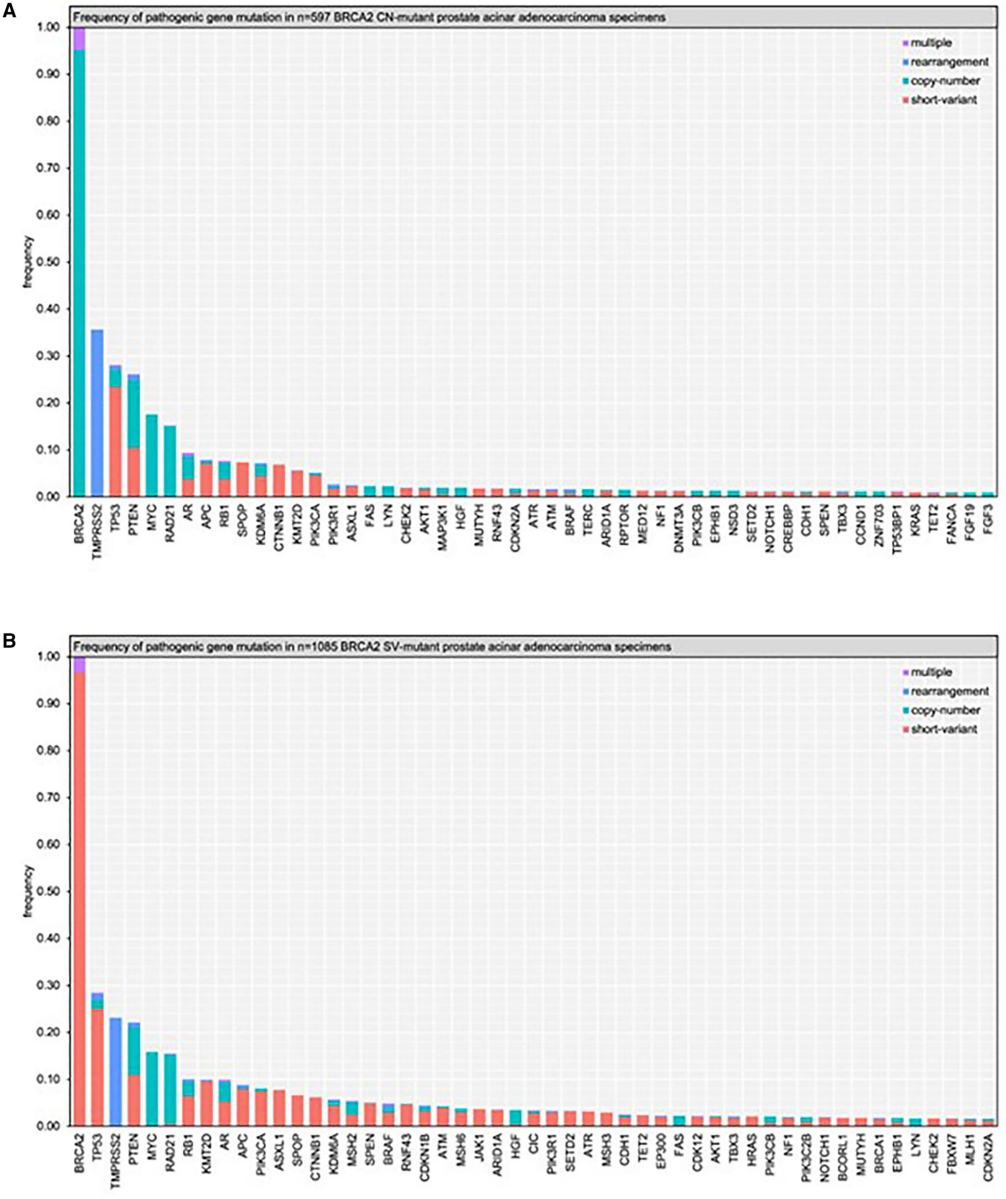
Bar chart showing the distribution of genomic alterations in clically-advanced Prostate Cancer (CAPC) cases with BRCA2-loss (2A) and BRCA2-short variant–mutated (svmut) (2B). Each graph shows the frequency of various types of genomic alterations, categorized as: short variant mutations, genomic copy number alterations, genomic rearrangements/fusions and multiple alterations per sample.

Figure 2 compares the distribution of genomic alterations in CAPC cases with BRCA2-loss and *BRCA2*-svmut groups.

### Computational somatic/germline predictions for pathogenic mutations

In the *BRCA2*-mut cohort, a total of 2582 pathogenic short variants were identified. Among these, 66.5% were classified as somatic and 21.3% as germline (Figure S1—see online supplementary material for a color version of this figure). The remaining 12.1% of the variants were of unknown origin. Regarding *BRCA2*-wt group, a total of 23 341 pathogenic short variants were identified. Of these, the majority—85.1%—were somatic, while only 5.7% of the variants were germline; an additional 10.2% of the variants remained unclassified.

## 4. Discussion

To our knowledge, this is one of the largest studies that evaluated the genomic landscape of CAPC in relation to HRDsig status, integrating comprehensive molecular data from over 22 000 tumor samples. Given the growing number of published and ongoing trials assessing the role of PARPi in biomarker-selected patients,^14–18^ our findings gain further importance as they demonstrate the importance of HRDsig as a potential predictor of PARPi efficacy, identifying a specific subgroup of patients with distinct genomic features and co-alterations that could possibly further guide personalized treatment strategies. Nevertheless, it should be emphasized that the predictive role of HRDsig in this setting has not yet been clinically validated, and its use for therapeutic decision-making cannot be recommended outside prospective studies.

PARP inhibitors exhibit their antitumor effects through a mechanism of synthetic lethality, which selectively affects cancer cells with impaired HRR mechanisms.^19^ In fact, under normal conditions, PARP enzymes play a key role in repairing single-strand DNA breaks (SSBs). However, when PARP is suppressed, these SSBs persist and eventually lead to the formation of more lethal double-strand breaks (DSBs) during DNA replication. In cells with a functional HRR pathway, DSBs can be efficiently repaired. By contrast, in cells lacking functional HRR key components, this repair process fails, resulting in accumulation of DNA damage that becomes lethal to the cancer cell.

As demonstrated in the TRITON2 trial, further supported by a large pan-cancer *BRCA1/2* analysis conducted by *Sokol et al*., bi-allelic inactivation seems to be more frequent in *BRCA2* than in *BRCA1* in prostate cancer.^20^^,^^21^ It is widely theorized that tumors harboring bi-allelic *BRCA1/2* loss exhibit particular sensitivity to PARPi, since the profound impairment of HRR pathways makes cancer cells highly dependent on alternative, error-prone DNA repair mechanisms. Supporting this hypothesis, recent evidence from *Triner D.* et al. demonstrated that among a cohort of 445 patients carrying HRR alterations, those harboring *BRCA 1/2* homozygous loss experienced more favorable clinical outcomes following treatment with PARPi compared to those with other deleterious *BRCA 1/2* alterations.^4^

In our study, HRDsig+ tumors were strongly enriched for *BRCA2* alterations, particularly bi-allelic deletions, further confirming the central role of *BRCA2* in driving HRD in CAPC. In contrast, consistent with previous reports,^21^  *BRCA1* alterations were extremely rare in our cohort, both in HRDsig+ and HRDsig− patients. It is important to highlight that nearly half of HRDsig+ tumors in our cohort did not harbor *BRCA2* mutations, suggesting that HRDsig captures a broader range of genomic instability mechanisms beyond single-gene alterations. This finding reinforces the potential clinical value of HRDsig as a complementary biomarker, possibly useful for selecting patients who may benefit from PARPi even in the absence of *BRCA* mutations. This hypothesis is particularly important when considering the current approval status of PARP inhibitors across different countries: in several regions, including parts of Europe and the United States, PARPi are currently approved primarily for patients with documented *BRCA1* or *BRCA2* mutation.^22^

Genomic ancestry distribution was overall comparable between groups. Although differences in ancestry distribution reached statistical significance, the absolute magnitude was small, and their biological relevance remains uncertain.

Another essential DDR mechanism for cell survival distinct from HRR is the MMR, that primarily corrects base-base mismatches and small insertion-deletion loops that occur during DNA replication.^23^ MSI is a molecular hallmark of defects in the MMR system, a condition that leads to genomic instability, ultimately contributing to an increased mutational burden and tumorigenesis. This provides a rationale for the clinical benefit observed with immune checkpoint inhibitors in patients with MSI-high or MMR-deficient tumors across several cancer types, including prostate cancer in selected cases.^24^

The genomic instability associated with both HRD and other DDR deficiencies, such as MMR-deficiency provides a strong rationale for evaluating PARPi plus ICI combinations.^25^ However, while preclinical evidence supports their potential synergistic effect, several clinical trials demonstrated that such combinations may not be effective in unselected patient populations.^26^ Despite interesting phase II trial results,^27^ in the phase III Keylink-010 randomized trial, the combination of pembrolizumab plus olaparib didn’t show a significant survival benefit in biomarker-unselected patients with mCRPC compared to androgen receptor pathway-inhibitors (ARPI), leading to a premature interruption of the trial.^28^

In our analysis, although MSI-high status was rare across all groups, it was significantly more frequent in *BRCA2*-svmut tumors (7.8%) compared to *BRCA2*-loss (0.2%) or *BRCA2*-wt (2.8%), suggesting that a subset of these tumors may harbor concurrent MMR deficiency. Similarly, TMB ≥ 20 mut/Mb was observed in 13.1% of BRCA2-svmut tumors vs. only 2.1% in *BRCA2*-loss and 3.9% in *BRCA2*-wt cases. Interestingly, MSI-high and TMB ≥ 20 mut/Mb were significantly more frequent in HRDsig− cases (*P* < .0001), as were MMR signatures (*P* < .0001).

These findings could suggest that *BRCA2*-svmut CAPC may harbor a broader range of genomic instability features beyond HRD alone, potentially identifying a subset of patients who could potentially benefit from PARPi + ICI combination strategies. In contrast, tumors with *BRCA2* loss or with HRDsig+ appear to be a more genomically defined subgroup, characterized by notable HRR impairment and lower rates of additional DNA repair defects, with a marked sensitivity to PARPi, but likely limited potential benefit from ICI. This might also reflect an evolutionary need: in tumors with such profound HRR deficiency, the acquisition of additional repair defects—such as MSI—could result in unsustainable levels of genomic instability, ultimately leading to cell death. In this context, the absence of additional repair pathway impairments may be necessary for tumor cell survival.

Beyond DDR mechanisms, our analysis revealed intricate co-occurrence patterns involving key tumor suppressors and oncogenic drivers, including TP53, RB1, PTEN, as well as alterations in MYC, RAD21, and SPOP. These findings align with mounting evidence that CAPC progression is shaped by complex molecular networks rather than isolated genomic events.^29^

In our cohort, *SPOP* mutated tumors appeared to be less frequent in HRDsig+ and in *BRCA*-loss cases compared to HRDsig− (*P* < .0001) and *BRCA*-wt (*P* = .047), respectively. *SPOP*-mutant CAPC have been previously associated with distinct molecular features, as well as enhanced sensitivity to ARPI.^30^ Interestingly, preclinical evidence suggests that *SPOP* inactivation could promote increased PARPi sensitivity.^31–33^ The low prevalence of *SPOP* mutations among HRDsig^+^ and *BRCA*-loss tumors observed in our analysis may possibly reflect a degree of mutual exclusivity between these two mechanisms, both of which appear to confer PARPi sensitivity through distinct molecular routes. This apparent mutual exclusivity raises important questions about the underlying biology: whether SPOP mutations and BRCA2 deficiency represent alternative evolutionary paths toward genomic instability, and whether their distinct mechanisms of PARPi sensitization could inform different therapeutic approaches or combination strategies.

Although a possible association between *ERG* and various components of DDR pathways has been partially investigated, its correlation with *BRCA* status remains less well defined.^34^  *Poulsen et al.* hypothesized that *TMPRSS2-ERG* fusions might represent a potential mechanism of resistance to PARPi, which is particularly intriguing considering that, in our study, this fusion was observed predominantly in the *BRCA2*-loss subgroup—patients who would theoretically be the most likely to benefit from PARPi inhibition. From a clinical point of view, these findings raise the possibility that co-occurring *TMPRSS2–ERG* fusions may potentially attenuate the efficacy of PARP inhibition, even in the presence of *BRCA2* loss. These findings further emphasize the importance of CGP beyond HRR genes to capture co-occurring alterations that may potentially influence treatment response. It should be further noted that *TMPRSS2-ERG* fusions in prostate cancer tend to be generally mutually exclusive with *SPOP* mutations.^30^

Our data also highlight important patterns involving *RB1* and *TP53* alterations, which carry critical implications for lineage plasticity and treatment resistance. Several studies have reported an association between *TP53/RB1* co-loss and the emergence of neuroendocrine prostate cancer (NEPC), a phenotype characterized by lineage plasticity, poor prognosis, and resistance to AR-targeted therapies.^35^ The combined inactivation of *RB1* and *TP53* has been shown to be sufficient to drive transdifferentiation toward a neuroendocrine phenotype in preclinical models, even in the absence of classical small-cell histology.^36^^,^^37^ This suggests that molecular profiling for *RB1* and *TP53* status may serve as a surrogate marker for neuroendocrine features and help identify patients at high risk of developing aggressive, AR-independent disease.

The relationship between HRD genes and NEPC biology, however, appears complex. *Aggarwal et al.* conducted a large multi-institutional prospective study in which metastatic biopsies were obtained from patients with progressive mCRPC after ARPI.^38^ In their cohort of 249 metastatic tumor samples, 27 were classified as treatment-emergent small-cell/neuroendocrine prostate cancer (t-SCNC). Interestingly, in these tumors, deleterious mutations and/or copy number losses in DDR pathway genes were found to be almost entirely mutually exclusive with the presence of t-SCNC.

Conversely, a recent systematic review reported that the pooled prevalence of deleterious *ATM/BRCA* mutations in NEPC was 16.8%, further suggesting a complex relationship between NEPC biology and HRD gene status.^35^ Moreover, when comparing genomic profiles, *de novo* NEPC showed a significantly higher frequency of concurrent *RB1/TP53* alterations, together with a numerically greater prevalence of *PTEN* loss and *ATM/BRCA* mutations, in contrast to what was observed in t-SCNC.

In our cohort, *RB1* alterations were more frequent in both *BRCA2-loss* (7.7%) and *BRCA2-svmut* (10.0%) tumors compared with *BRCA2*-wt (4.6%), supporting the hypothesis that *BRCA2* deficiency may predispose to neuroendocrine features (*P* = .003 and *P* < .001, respectively). In contrast, *TP53* alterations were less frequent in *BRCA2*-deficient tumors (29.0%) than in *BRCA2*-wt (39.2%; *P* < .001). HRDsig+ tumors also carried higher rates of *RB1* alterations, reinforcing the possible connection between HRD and neuroendocrine features.

In the context of BRCA2-altered tumors, it would be particularly informative to investigate whether the co-occurrence of RB1 and TP53 loss is associated with a higher incidence of NEPC. This has important therapeutic implications: patients with dual *RB1/TP53* loss may derive limited benefit from PARPi monotherapy and could instead require platinum-based chemotherapy, which has demonstrated efficacy in NEPC.^39^ Unfortunately, it should be noted that patients classified as NEPC within the FMI database were not included in the present analysis, which limits the possibility of directly correlating our genomic findings with histologically confirmed NEPC. Despite this limitation, the molecular identification of combined *RB1/TP53* alterations in CAPC may indicate biologically neuroendocrine behavior, making the interpretation of our findings in the context of the existing literature more challenging.

The enrichment of *MYC* amplifications in HRDsig+ tumors also is particularly interesting, as *MYC* overexpression has been implicated in driving aggressive disease phenotypes.^40^ When stratified by *BRCA2* status, *MYC* alterations were significantly more frequent in both *BRCA2-loss* and *BRCA2-svmut* compared to *BRCA2-wt* tumors (*P* < .001 for both comparisons), suggesting a specific co-occurrence pattern between *BRCA2* deficiency and *MYC* amplification. *MYC* may cooperate with *BRCA2* deficiency to accelerate tumor progression through enhanced replication stress and chromosomal instability.

Similarly, the higher frequency of *RAD21* alterations in HRDsig+ cases, as well as in BRCA2-loss and BRCA2-svmut compared with BRCA2-wt, suggests a convergence of defects affecting both homologous recombination and chromatin structural integrity. RAD21, a core component of the cohesin complex, plays essential roles in sister chromatid cohesion, DNA repair, and transcriptional regulation.^41^ Its alteration in the context of pre-existing HRD may further compound genomic instability and potentially influence therapeutic responses.

Several limitations need to be considered. First, the retrospective nature of the study may have introduced selection and confounding biases, while the absence of treatment and clinical outcomes data restricted our ability to directly correlate GA with treatment response and survival. In addition, we did not have adequate information on the extent (eg, stage, tumor sites) of disease at the time of tissue sampling and whether or not there was castration resistance present. Furthermore, Gleason score and PSA values were not available in the FMI CGP dataset, which primarily contains molecular and limited demographic information, precluding integration of these important clinical–pathological parameters with our genomic findings. Moreover, our study was focused on tissue CGP, while future work should also include plasma ctDNA, also integrating detailed clinicopathologic information, treatment history, disease burden, and survival outcomes to better define the relationship between HRD and outcomes for patients with CAPC.

A further limitation concerns the HRDsig cutoff of ≥0.7, which was originally derived from a pan-cancer training set to achieve high sensitivity for detecting biallelic *BRCA1/2* alterations.^6^ The foundational study by Moore et al. did not report prostate-specific optimization of this threshold, and alternative cutoffs were not explored in that analysis. Consequently, it remains uncertain whether ≥0.7 represents the optimal discriminator in clinically advanced prostate cancer, where the genomic context of HRD may differ from that of breast or ovarian malignancies. In the present study we applied the predefined cutoff without re-calibration, and no sensitivity analyses using alternative thresholds were performed. Prospective studies specifically focused on prostate cancer will be required to refine HRDsig thresholds and to validate their predictive value for PARPi response in CAPC.

## 5. Conclusions

Our hypothesis-generating results support the need for CGP enabling more inclusive biomarker criteria exploration. Incorporating HRD signatures as companion diagnostics may possibly allow more precise patient selection and better trial designs.

## Supplementary Material

oyag100_Supplementary_Data

## Data Availability

The datasets generated and/or analyzed during the current study are not publicly available because they are proprietary to Foundation Medicine and subject to confidentiality restrictions, but are available from the corresponding author on reasonable request.

## Prior presentations

This work has been previously presented by C.M. in part as follows:

- **Poster presentation** at the 2024 ESMO Annual Meeting (Barcelona, Spain; September 15, 2024), abstract titled: “*1613P: Clinically Advanced Prostate Cancer (CAPC) Featuring BRCA2 Loss: A Comprehensive Genomic Profiling (CGP) Study*”.
- **Poster presentation** at the *2025 Genitourinary Cancers Symposium* (San Francisco, CA; January 30, 2025), abstract titled: *“Homologous recombination deficiency signature (HRDsig) and the genomic landscape of clinically advanced prostate carcinoma (CAPC)”*. This presentation received a *Conquer Cancer Merit Award*.
