# Supplementary material for: Homologous recombination deficiency and genomic alterations in advanced prostate cancer: insights for precision therapy
Source: Oncologist. 2026 Mar 24;31(4):oyag100. doi: 10.1093/oncolo/oyag100 (PMC13253569; doi:10.1093/oncolo/oyag100)
Supplement: oyag100_Supplementary_Data [file oyag100_supplementary_data.docx]

Supplementary figure 1A


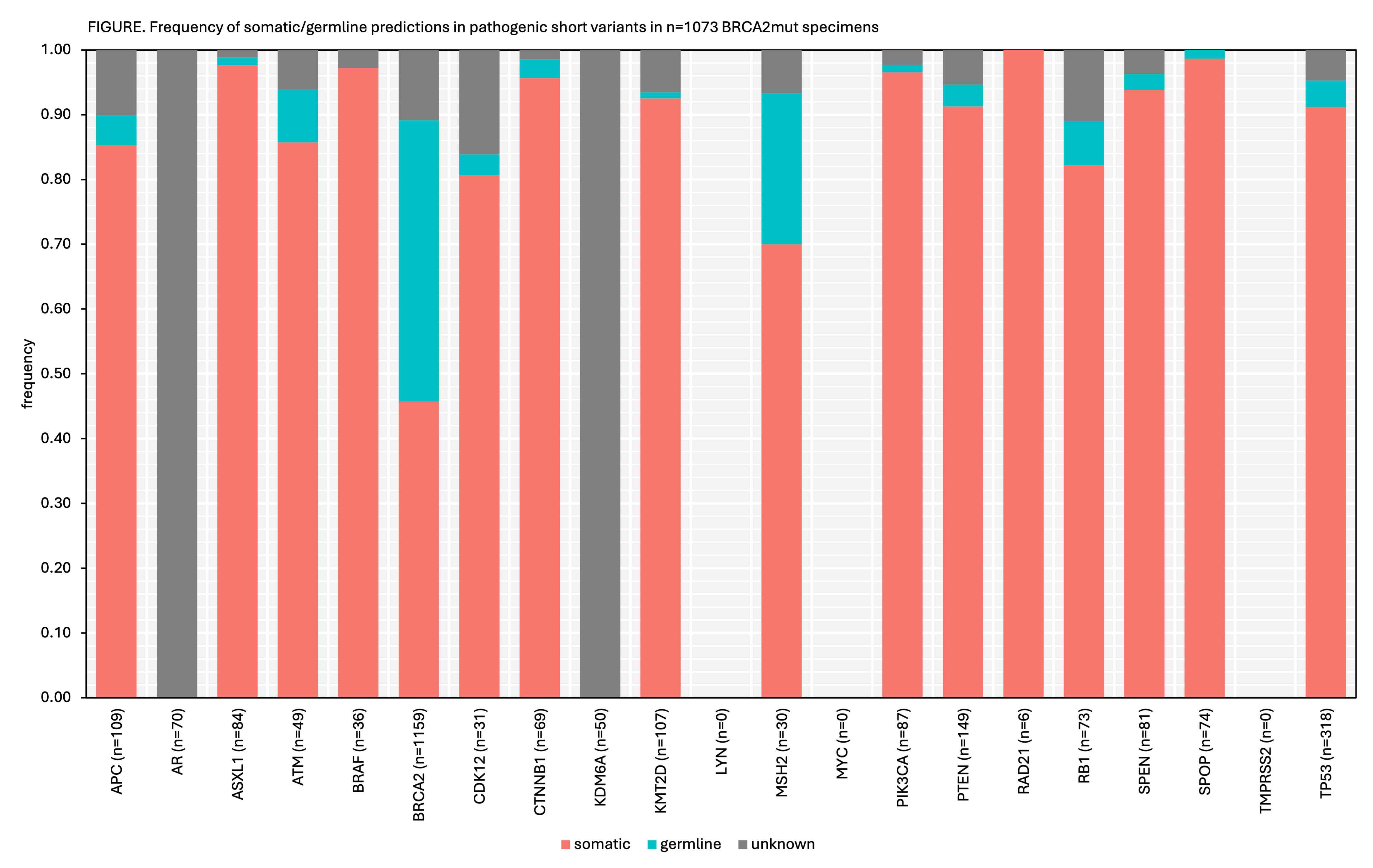


Supplementary figure 1B


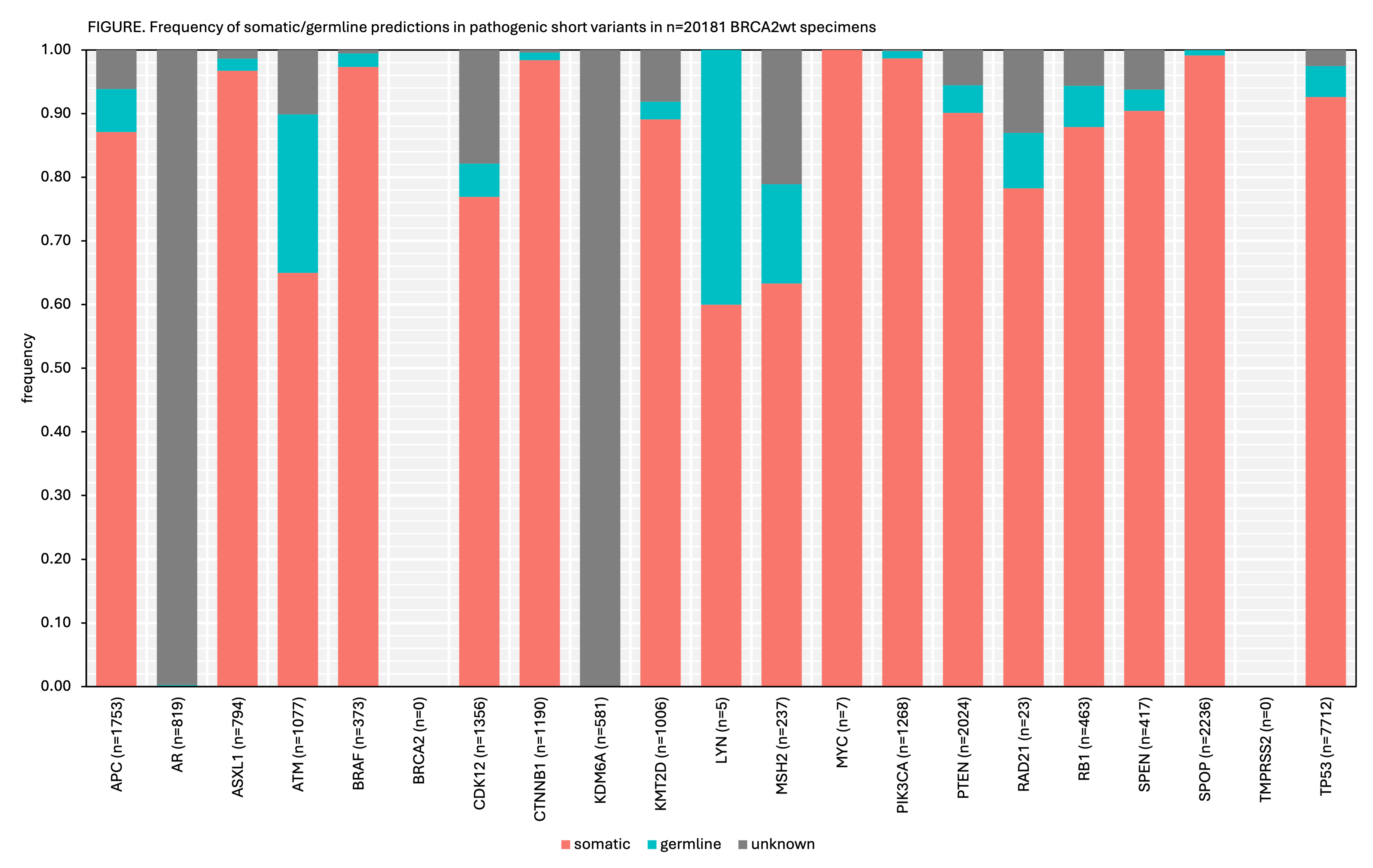


Supplementary figure 1:

Computational somatic/germline predictions for pathogenic mutations in our list of genes within the BRCA2-mut (n=1073; 1A) and BRCA2-wt (n=20181; 1B) specimens.

Red=somatic; blue=germline; grey=unknown
